# Supplementary material for: Formation of robust bound states of interacting microwave photons
Source: Nature. 2022 Dec 7;612(7939):240–5. doi: 10.1038/s41586-022-05348-y (PMC9729104; doi:10.1038/s41586-022-05348-y)
Supplement: Supplementary file 1 — Supplementary Sections 1–7, Figs. 1–16 and References. [file 41586_2022_5348_MOESM1_ESM.pdf]

nature portfolio

https://doi.org/10.1038/s41586-022-05348-y

Supplementary information

Formation of robust bound states of interacting microwave photons

In the format provided by the authors and unedited

Supplement - Formation of robust bound states of interacting photons

Google Quantum AI and Collaborators
(Dated: September 2, 2022)

Contents

I. Quantum processor details and coherence times 2

II. 2-Qubit fSim gates 2
A. fSim calibration 2
B. fSim gate control error 4

III. Analytical results for the Floquet XXZ chain 6
A. Band structure 6
B. Group velocity 6
C. Choice of parameters θ and ϕ 7

IV. Examples of multi-photon spectroscopy 8

V. Supplementary data 10
A. Trajectories 10
B. Spectroscopy 11

VI. Integrability of the Floquet XXZ model 15

VII. Numerical analysis of the breaking of integrability 17
A. Level statistics 17
B. Stability of the $n_{\text{ph}} = 3$ domain wall 17

References 19

I. Quantum processor details and coherence times

The experiment is performed on a quantum processor with similar design as that in Ref. [1]. The qubits are transmons with tunable frequencies and interqubit couplings. Figure S1a shows the single-qubit relaxation times of the 24 qubits used in the experiment, where a median value of $T_1 = 16.1$ µs is found. The dephasing times T_2^*, measured via Ramsey interferometry, are shown in Fig. S1b and have a median value of 5.3 µs. Lastly, The T_2 values after CPMG dynamical decoupling sequences are also shown in Fig. S1b and have a median of 17.8 µs.

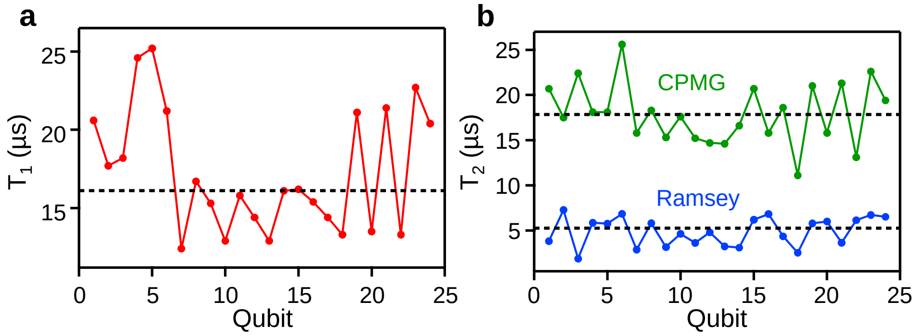

FIG. S1. **a,** Single-qubit relaxation times T_1 across the 24-qubit ring. Dashed line represents the median value of 16.1 µs. **b,** Single-qubit T_2 values measured via Ramsey and Carr-Purcell-Meiboom-Gill (CPMG) dynamical decoupling sequence. Dashed lines indicate median T_2 values of 5.3 µs (Ramsey) and 17.8 µs (CPMG), respectively.

II. 2-Qubit fSim gates

A. fSim calibration

The floquet unitaries used in the experiment are composed of alternating layers of fSim(θ, ϕ, β) gates which are defined as:

$$\text{fSim}(\theta,\phi,\beta) = \begin{pmatrix} 1 & 0 & 0 & 0 \\ 0 & \cos\theta & ie^{i\beta}\sin\theta & 0 \\ 0 & ie^{-i\beta}\sin\theta & \cos\theta & 0 \\ 0 & 0 & 0 & e^{i\phi} \end{pmatrix}, \tag{S1}$$

where θ is the SWAP angle and ϕ is the conditional phase, and β is phase accumulated resulting from hopping. For open chains, β is not gauge invariant and can be ignored, but for closed chains, non-zero β values lead to a total flux threading the closed chain. fSim(θ, ϕ, β) describes the unitary form output by a DC pulse bringing the fundamental frequencies ω_1 and ω_2 of two transmons into resonance and turning on their interqubit coupling g for a given time duration t_p, as illustrated in Fig. S2a. During t_p, resonant interaction between the $|10\rangle$ and $|01\rangle$ states of the two transmons leads to population transfer and a finite θ. Additionally, dispersive interaction between the $|11\rangle$ and $|02\rangle$ (as well as $|20\rangle$) states of the two qubits gives rise to a finite conditional phase ϕ.

Due to the frequency detunings of the qubits during the DC pulse, the fSim unitary also includes additional single-qubit Z rotations and is more generally described by:

$$\text{FSIM}(\theta,\phi,\gamma,\alpha,\beta) = \begin{pmatrix} 1 & 0 & 0 & 0 \\ 0 & e^{i(\gamma-\alpha)}\cos\theta & ie^{i(\gamma+\beta)}\sin\theta & 0 \\ 0 & ie^{i(\gamma-\beta)}\sin\theta & e^{i(\gamma+\alpha)}\cos\theta & 0 \\ 0 & 0 & 0 & e^{2i\gamma}e^{i\phi} \end{pmatrix}, \tag{S2}$$

where γ, α and β are complex phases incurred by the single-qubit Z rotations. These single-qubit phases are calibrated and reduced to nearly zero using the technique of Floquet calibration described in our previous works [2–4]. Here we focus on the tuning and calibration of the two-qubit angles θ and ϕ.

Figure S2c and Figure S2d show experimentally obtained values of θ and ϕ as functions of pulse parameters t_p and g_{max}. In these measurements, the technique of unitary tomography [5] is used to estimate the angles. We have also enforced a Gaussian filter with time constant 5 ns on the rising/falling edges of the pulse on g to ensure adiabatic

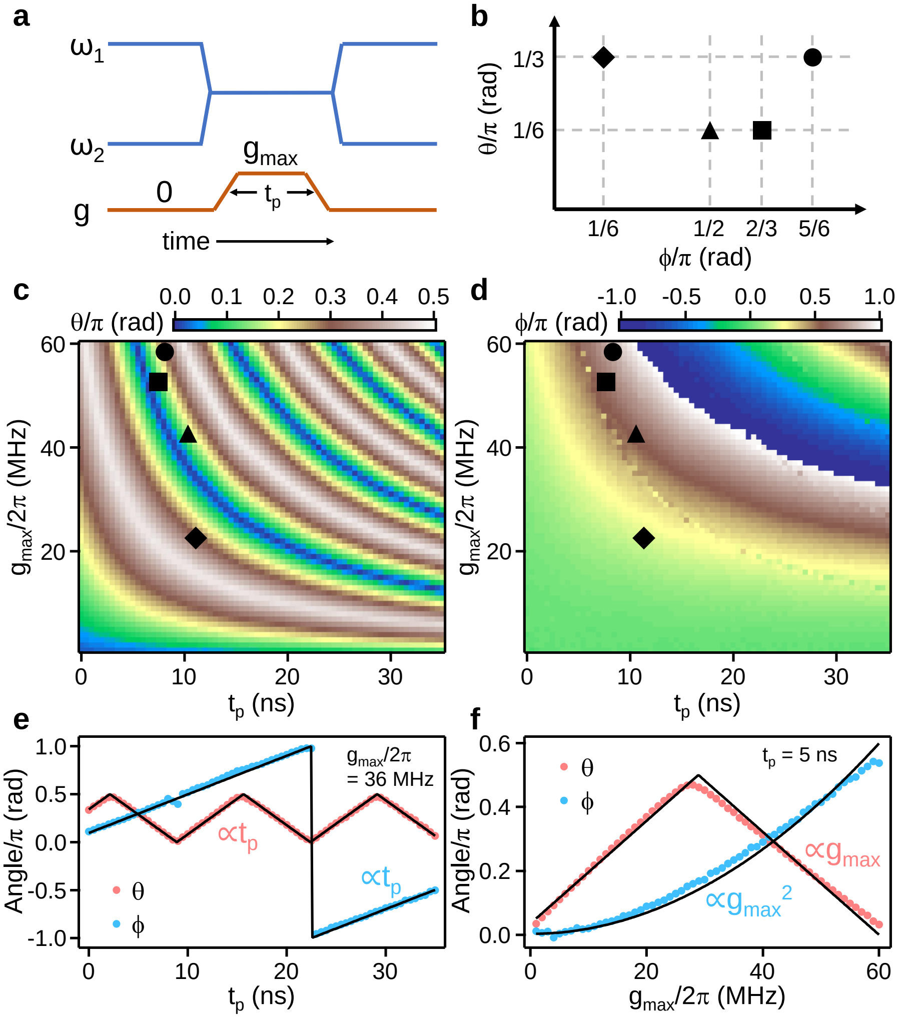

FIG. S2. **a,** Schematic illustration of the DC pulse used to realize a fSim gate. **b,** Schematic plot showing the four sets of fSim angles used in this work: $(\theta/\pi, \phi/\pi) = (\frac{1}{3}, \frac{5}{6})$ (filled circle), $(\frac{1}{6}, \frac{2}{3})$ (square), $(\frac{1}{6}, \frac{1}{2})$ (triangle) and $(\frac{1}{3}, \frac{1}{6})$ (diamond). **c,** Experimentally measured θ as a function of pulse length t_p and maximum interqubit coupling g_{max}. The approximate pulse parameters for the fSim gates in panel b are indicated by their corresponding symbols. **d,** Similar plot as panel c but with ϕ shown instead. **e,** θ and ϕ as functions of t_p for a fixed $g_{max}/2\pi$ of 36 MHz. Solid lines show linear fits. **f,** θ and ϕ as functions of g_{max} for a fixed t_p of 5 ns. Solid lines show a linear fit $\propto g_{max}$ to $\theta(g_{max})$ and a quadratic fit $\propto g_{\mathrm{max}}^2$ to $\phi(g_{max})$.

evolution with respect to the $|11\rangle \rightarrow |20\rangle$ and $|11\rangle \rightarrow |02\rangle$ transitions. This is important to minimize leakage. We observe that θ shows a series of maxima/minima corresponding to values of t_p and g_{max} where $|01\rangle$ is fully transformed to $|10\rangle$ or returned back to $|01\rangle$. On the other hand, ϕ increases monotonically over t_p and g_{max} until it reaches a maximum value of π where it is wrapped by 2π and becomes $-\pi$. Given the dependence of both unitary angles on both pulse parameters t_p and g_{max}, independent control of θ or ϕ is not possible with a single pulse parameter. As such, past works have added a resonant pulse between the $|11\rangle$ and $|02\rangle$ states to enact a pure CPHASE gate, thereby enabling full tunablity over θ and ϕ [5]. The additional pulse, however, significantly increases the complexity of quantum control and is also prone to leakage.

In this work, we have chosen to perform fSim gates directly using the single pulse in Fig. S2a. Our approach relies on the different scaling of θ and ϕ with the pulse parameters, as illustrated by Fig. S2e and Fig. S2f. Here we observe that while θ and ϕ both scale linearly with t_p, the scaling with g_{max} is different for the two angles: whereas θ scales linearly with g_{max}, $\phi \propto g_{\mathrm{max}}^2$ due to the fact that dispersive shift of the $|11\rangle$ state by the $|02\rangle$ and $|20\rangle$ states is proportional to g^2/Δ, where Δ is the frequency difference between $|11\rangle$ and the $|02\rangle$ ($|20\rangle$). The difference in scaling implies that it is possible to achieve a desired combination of θ and ϕ by choosing a particular "contour" in the 2D space of (t_p, g_{max}) where θ has the target value, then increasing (decreasing) g_{max} while decreasing (increasing) t_p until ϕ attains the target value as well.

Practically, ϕ and θ are calibrated via a simple gradient descent method: we start with an initial guess (t_0, g_0) for the pulse parameters (t_p, g_{max}) based on the 2D scan shown in Fig. S2c and Fig. S2d. The corresponding values of ϕ

and θ are then accurately determined via Floquet calibration [2–4] which we denote as ϕ_0 and θ_0. We then calibrate the fSim angles at $(t_{\mathrm{p}}, g_{max}) = (t_0 + \delta t, g_0)$ and $(t_{\mathrm{p}}, g_{\mathrm{max}}) = (t_0, g_0 + \delta g)$. The results allow us to approximate the following gradient matrix:

$$M_{\mathrm{g}} = \begin{pmatrix} \partial\phi/\partial t & \partial\phi/\partial g \\ \partial\theta/\partial t & \partial\theta/\partial g \end{pmatrix}. \tag{S3}$$

A new set of pulse parameters (t_1, g_1) are then computed from the gradient matrix and the deviations from target fSim angles, $(\Delta t, \Delta g) = (t_{\mathrm{c}} - t_0, g_{\mathrm{c}} - g_0)$, via:

$$\begin{pmatrix} t_1 \\ p_1 \end{pmatrix} = M_{\mathrm{g}}^{-1} \begin{pmatrix} \Delta t \\ \Delta g \end{pmatrix} + \begin{pmatrix} t_0 \\ p_0 \end{pmatrix}. \tag{S4}$$

The fSim angles $(t_{\mathrm{p}}, g_{max})$ are then measured at the new pulse parameters (t_1, g_1) and the process is repeated. Generally only two gradient descents are sufficient to reach control errors on the level of ∼20 mrad for both θ and ϕ.

When repeating the fSim gate n times in Floquet calibration, the error accumulates as:

$$\mathrm{fSim}(\theta,\phi) = \begin{pmatrix} 1 & 0 & 0 & 0 \\ 0 & e^{i\gamma n}A & ie^{i(\gamma n+\beta)}B & 0 \\ 0 & ie^{i(\gamma n-\beta)}B & e^{i\gamma n}A^* & 0 \\ 0 & 0 & 0 & e^{2i\gamma n}e^{i\phi n} \end{pmatrix} \tag{S5}$$

with

$$A = \cos n\Omega - iQ\sin n\Omega \tag{S6}$$
$$B = \sqrt{1-Q^2}\sin n\Omega \tag{S7}$$
$$\cos\Omega = \cos\theta\cos\alpha/2 \tag{S8}$$
$$Q = \cos\theta\sin\alpha/2/\sin\Omega \tag{S9}$$

B. fSim gate control error

The angular errors, which are measured using periodic Floquet calibration as outlined in section II A, are displayed in Fig. II A along with the measured 2-qubit Pauli error for the four pairs of θ,ϕ studied in our work. The angular errors of the fSim gate can be combined into an overall control error by calculating the coherent gate infidelity:

$$\epsilon_c = 1 - F = \frac{1}{1+D^{-1}} \overbrace{\left(1 - \frac{1}{D^2}|\mathrm{Tr}\{U^\dagger_{\mathrm{target}}U_{\mathrm{actual}}\}|^2\right)}^{e_{\mathrm{P}}} \tag{S10}$$

where e_{P} is the Pauli error, $D = 4$ is the size of the computational subspace of the gate, and $U_{\mathrm{target(actual)}}$ is the target (actual) unitary. Inserting the unitary matrix in eq. S2, we find the control error in terms of the angular errors:

$$\epsilon_c = \frac{2}{5}d\theta^2 + \frac{3}{20}d\phi^2 + \frac{1}{10}d\gamma^2 + \frac{1}{5}d\gamma d\phi + \frac{1}{10}\cos^2\theta\cdot d\alpha^2 + \frac{1}{80}\sin^2\theta\left(7+\cos 2\theta + 2\sin^2\theta\right)\cdot d\beta^2 \tag{S11}$$

For the four angle pairs in our study, we find median control errors of $\epsilon_c(\theta = \pi/6, \phi = 2\pi/3) = 1.5\cdot 10^{-4}$, $\epsilon_c(\theta = \pi/6, \phi = \pi/2) = 1.0\cdot 10^{-4}$, $\epsilon_c(\theta = \pi/3, \phi = 5\pi/6) = 3.1\cdot 10^{-4}$, and $\epsilon_c(\theta = \pi/3, \phi = \pi/6) = 1.8\cdot 10^{-4}$.

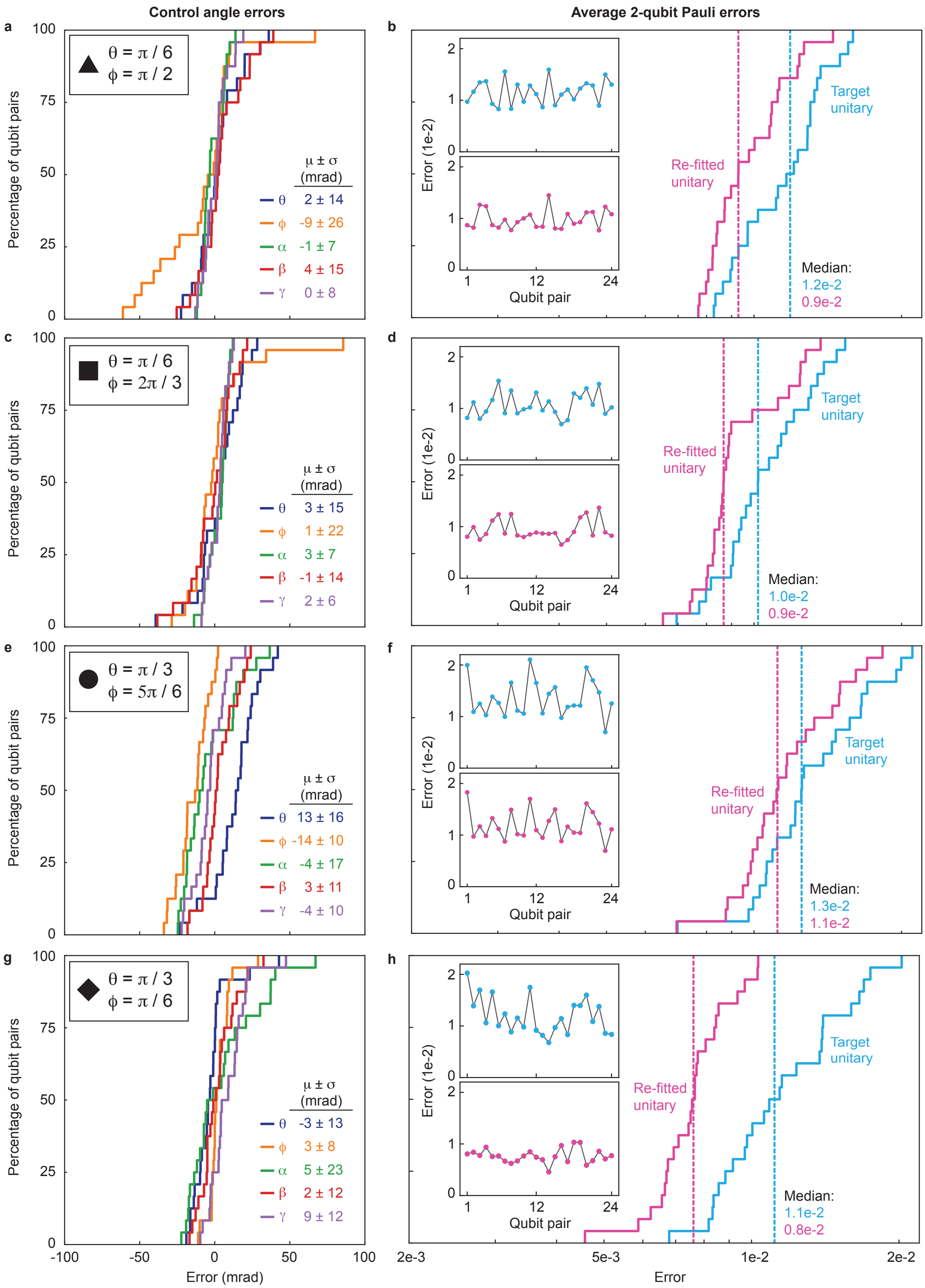

FIG. S3. **a**, Integrated histogram of errors in the control angle parameters of the fSim gate as measured by periodic calibration, for target angles $\theta = \pi/6$, $\phi = \pi/2$, $\alpha = \beta = \gamma = 0$. Inset: table displaying the mean errors and their standard deviation. **b**, Integrated histogram of average 2-qubit Pauli errors, considering both the target unitary (blue) and the re-fitted unitary (pink). Inset: Pauli errors plotted against qubit position, demonstrating the absence of systematic spatial dependence. Dashed vertical lines denote the medians. **c-h**, Same as **a**,**b**, but for target fSim angles, $\theta = \pi/6$, $\phi = 2\pi/3$ (**c**,**d**), $\theta = \pi/3$, $\phi = 5\pi/6$ (**e**,**f**), $\theta = \pi/3$, $\phi = \pi/6$ (**g**,**h**). The remaining target angles are zero ($\alpha = \beta = \gamma = 0$) in all plots.

III. Analytical results for the Floquet XXZ chain

A. Band structure

In this section, we present analytical expressions for the dispersion relations in the Floquet XXZ model, as derived in Ref.[6]. The solution is separated into two cases, corresponding - in the limit of the Hamiltonian model - to the gapped ($\phi > 2\theta$) and the gapless ($\phi < 2\theta$) phase.

We first focus on the gapped phase ($\phi > 2\theta$), since in this case, there exists a bound state for all $n_{\rm ph}$ for every momentum (see end of section 3.5 p.19 in Ref.[6]). Importantly, the dispersion relations of the bound states have a similar functional form to that of a single particle excitation (see Eqs. (72) and (85) in [6]):

$$\cos(E(k) - \chi) = \cos^2(\alpha) - \sin^2(\alpha)\cos(k) \quad \text{with} \quad k \in [-\pi, \pi], \tag{S12}$$

where α and χ depend on the number of photons:

- For $n_{\rm ph} = 1$, the parameters are $\alpha = \theta$ and $\chi = 0$
- For $n_{\rm ph} \geq 2$, one finds:

$$\chi = n_{\rm ph}\phi - 2\arctan\left(\tan(\phi/2)\tanh\eta\coth(n_{\rm ph}\eta)\right) \tag{S13}$$

$$\cos^2\alpha = \frac{\cos^2\theta\sinh^2 n_{\rm ph}\eta}{\cos^2\theta\sinh^2 n_{\rm ph}\eta + \sin^2\theta\sinh^2\eta}, \tag{S14}$$

where η is given by:

$$\sinh^2(\eta) = \frac{\cos^2\theta - \cos^2(\phi/2)}{\sin^2\theta}. \tag{S15}$$

In the gapless regime ($\phi < 2\theta$), the bound state is only predicted to appear for one of the two branches in the first Brillouin zone and for a finite range of momenta, with limits given by (see end of section 3.5 p.19 in Ref.[6]):

$$k_0 = \pm 2n_{\rm ph}\eta \tag{S16}$$

These are the limits plotted with dashed black vertical lines in Fig. 3d in the main text, as well as in Fig. S8d. In this case the dispersion relation is similar to the gapless case with the substitution $\sinh\eta \mapsto \sin\eta$ and $\tanh\eta \mapsto \tan\eta$.

B. Group velocity

We can calculate the group velocity by differentiating both sides of S12:

$$v_g(k) = \frac{\mathrm{d}E}{\mathrm{d}k} = -\frac{\sin^2\alpha\sin k}{\sin(E(k) - \chi)} \tag{S17}$$

We notice that the maximum group velocity is achieved for $k = \pi/2$ leading to

$$v_g^{\rm max} = \frac{\sin^2\alpha}{\sqrt{1 - \cos^4\alpha}} \tag{S18}$$

This is the velocity of the initial cone that is shown in the main text Fig.2d

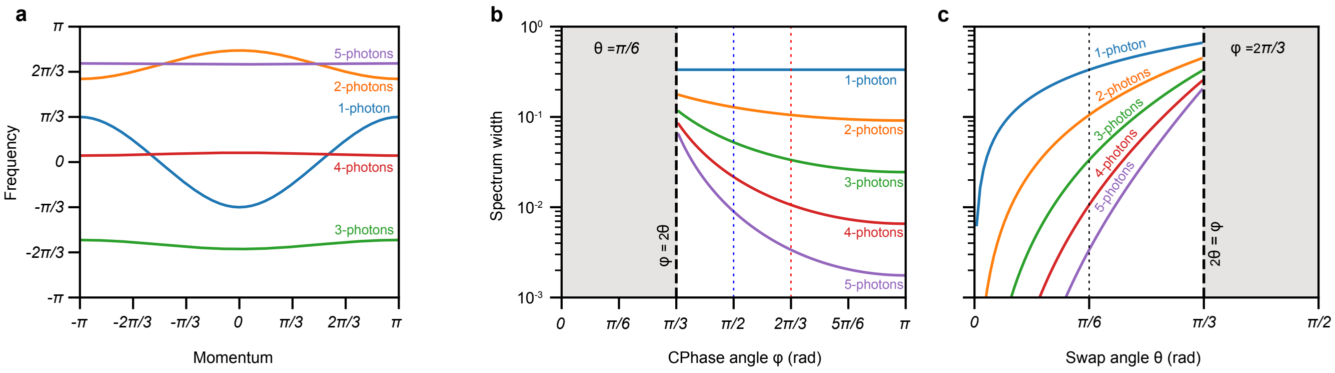

FIG. S4. **a**, Band structure of the different bound states for $\theta = \pi/6$ and $\phi = 2\pi/3$. **b, c**, Width of the $n_{\rm ph}$-photon band structure as a function of the parameters of the fSim gate. All these results are calculated using the exact solution given in eq. S12.

C. Choice of parameters θ and ϕ

The choice of the angles θ and ϕ of the fSim gate is dictated by several considerations, with regards to both the physics of the bound state and the experimental parameters of the fSim gate. In the main text, we present several angles as they have differents properties:

- First and foremost, the behavior is significantly different for the gapped $\phi > 2\theta$ regime and the gapless regime. We have predominantly focused on the gapped regime where bound states exist for all momenta, simplifying the analysis.
- Secondly, as shown in Fig. S4b, the amount of dispersion in the spectrum increases with increasing (decreasing) values of θ (ϕ). Hence, the ratio ϕ/θ should not be too large in order to maximize the visibility of the dispersion.
- Higher values of ϕ cause the bound state to be more localized, as intuitively expected from the stronger interactions.
- Finally the quality of the fSim gate depends on the angles, since strong interaction requires longer gates time or larger interaction strength (see section II).

IV. Examples of multi-photon spectroscopy

In this section we provide a pedagogical illustration of the basics of multi-photon spectroscopy by stepping through two simple examples. We focus on Hamiltonian dynamics, which is conceptually similar to the Floquet dynamics. The Fourier transform in space that was done in the main text is resulting from the space invariance of the circuit. For simplicity we only focus on the time-energy transformation and do not talk about the space-momentum transformation.

Consider a Hamiltonian $\hat{\mathcal{H}}$ with eigenenergies ω_n and eigenstates $|\varphi_n\rangle$

$$\hat{\mathcal{H}}\,|\varphi_n\rangle = \omega_n\,|\varphi_n\rangle\,. \tag{S19}$$

We seek a dynamical approach to learn the spectrum of $\mathcal{H}$. Consider an initial state $|\psi_0\rangle$ and its evolution $|\psi_t\rangle$

$$|\psi_0\rangle = \sum_n c_n\,|\varphi_n\rangle\,, \qquad |\psi_t\rangle = e^{-i\hat{\mathcal{H}}t}\,|\psi_0\rangle = \sum_n c_n\,e^{-i\omega_n t}\,|\varphi_n\rangle\,, \tag{S20}$$

where the complex coefficient $c_n = \langle\varphi_n|\,\psi_0\rangle$ is the overlap of $|\psi_0\rangle$ with $|\varphi_n\rangle$. The desired spectrum of $\hat{\mathcal{H}}$ can be extracted (e.g. by Fourier transform) from the overlap of $|\psi_0\rangle$ and $|\psi_t\rangle$

$$\langle\psi_0|\,\psi_t\rangle = \sum_n |c_n|^2\,e^{-i\omega_n t}\,. \tag{S21}$$

This overlap also frequently appears in non-equilibrium dynamics questions. Next, we provide a scheme to computing it via two simple examples.

Single-excitation example. We begin with the case of single photon dynamics in a 4-qubit system. We are only considering excitation conserving Hamiltonians. The resulting unitary $\hat{U}$ when written in the basis of $|0000\rangle\,,|1000\rangle\,,|0100\rangle\,,|0010\rangle$ and $|0001\rangle$ becomes:

$$\begin{array}{ccccc} |0000\rangle & |1000\rangle & |0100\rangle & |0010\rangle & |0001\rangle \end{array}$$
$$\hat{U} = \begin{pmatrix} 1 & 0 & 0 & 0 & 0 \\ 0 & u_{11} & u_{12} & u_{13} & u_{14} \\ 0 & u_{21} & u_{22} & u_{23} & u_{24} \\ 0 & u_{31} & u_{32} & u_{33} & u_{34} \\ 0 & u_{41} & u_{42} & u_{43} & u_{44} \end{pmatrix}. \tag{S22}$$

The first insight is using the fact that $\hat{U}$ is photon conserving and the $|0000\rangle$ manifold is detached from the other manifolds. We leverage this fact and create a phase-sensitive initial state (normalizations ignored)

$$|\psi_0\rangle = |0000\rangle + |0100\rangle\,, \tag{S23}$$

which leads to

$$|\psi_t\rangle = \hat{U}\,|\psi_0\rangle = |0000\rangle + u_{12}\,|1000\rangle + u_{22}\,|0100\rangle + u_{32}\,|0010\rangle + u_{42}\,|0001\rangle\,. \tag{S24}$$

From equations S19 to S21 it is evident that the spectrum is *always* given by the Fourier transform of $\langle\psi_0|\,\psi_t\rangle$. The task is to find an observable which is a measure of this overlap. Given the initial state we have chosen, then $\langle\psi_0|\,\psi_t\rangle = 1+u_{22}$. The second insight is to measure a non-hermitian operator $\hat{M}_{Q2} \equiv |0000\rangle\,\langle 0100|$, because:

$$\langle\psi_t|\,\hat{M}_{Q2}\,|\psi_t\rangle = \langle\psi_t|\,u_{22}\,|0000\rangle = u_{22}. \tag{S25}$$

Note that the qubit lowering operator acting on Q2, σ_2^-, could also be written as

$$\sigma_2^- = \hat{X}_2 - i\hat{Y}_2 = \hat{M}_{Q2} + |....\rangle\,\langle\text{more than single excitation}|. \tag{S26}$$

Since $|\psi_t\rangle$ has terms with only single photons, all gray colored terms can be ignored, and the Fourier transform of $\langle\psi_t|\,\sigma_2^-\,|\psi_t\rangle$ provides the desired spectrum. Notably, this would not have been achieved if the initial state was $|\psi_0\rangle = |0100\rangle$. While the overlap $\langle\psi_0|\,\psi_t\rangle = u_{22}$ still gives the spectrum, we find $\langle\psi_t|\,\hat{M}_{Q2}\,|\psi_t\rangle = 0$ and also $\langle\psi_t|\,\sigma_2^-\,|\psi_t\rangle = 0$, which clearly does not allow for measuring u_{22}.

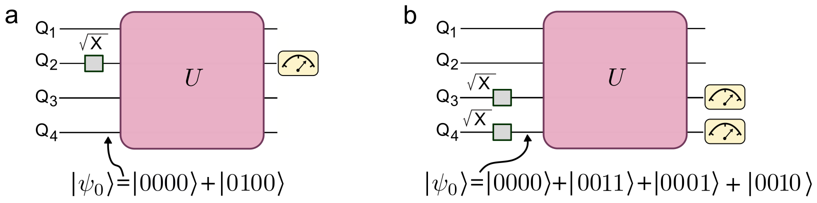

FIG. S5. Pulse sequences for determining the spectra of **a,** a single excitation, and **b,** two excitations for 4 qubits.

Multi-excitation example. Next, we consider the photon conserving dynamics of two excitations in a 4 qubit system, with the computational basis $|1100\rangle, |1010\rangle, |1001\rangle, |0011\rangle, |0101\rangle, |00110\rangle$. We start by placing Q3 and Q4 in a $|0\rangle + |1\rangle$ superposition, which gives rise to a superposition of the vacuum and a two-excitation state, as well as undesired single excitation states:

$$|\psi_0\rangle = |0000\rangle + |0011\rangle + |0001\rangle + |0010\rangle . \tag{S27}$$

The appearance of single excitation terms is not desired and could have been avoided by using proper entangling gates to arrive at $|0000\rangle + |0011\rangle$ as a more relevant initial state. However, as our experimental results show, populating (wrong) manifolds with fewer number of excitations, is not harmful since the two-qubit lowering operator does not couple these manifolds to the vacuum. The existence of these undesired states does, however, reduce the signal contrast, due to distributed probabilities. The evolution of the initial states results in:

$$\hat{U}\,|\psi_0\rangle = |0000\rangle + u_{14}\,|1100\rangle + u_{24}\,|1010\rangle + u_{34}\,|1001\rangle + u_{44}\,|0011\rangle + u_{54}\,|0101\rangle + u_{64}\,|0110\rangle + |\text{single excitation bitstrings}\rangle . \tag{S28}$$

The two-qubit lowering operator acting on Q3 and Q4, can also be written as

$$\sigma_3^- \sigma_4^- = \hat{X}_3\hat{X}_4 - \hat{Y}_3\hat{Y}_4 + i\hat{X}_3\hat{Y}_4 + i\hat{Y}_3\hat{X}_4 = |0000\rangle\,\langle 0011| + |....\rangle\,\langle \text{more than two excitations}| . \tag{S29}$$

Hence, the presence of terms in other manifolds does not leading to wrong answers and $\langle\psi_t|\,\sigma_3^-\sigma_4^-\,|\psi_t\rangle$ provides the desired 2-photon spectrum. The number of independent Pauli strings that need to be measured scales exponentially with the number of excitations in the manifold, and hence this method is not scalable to large photon number systems.

V. Supplementary data

A. Trajectories

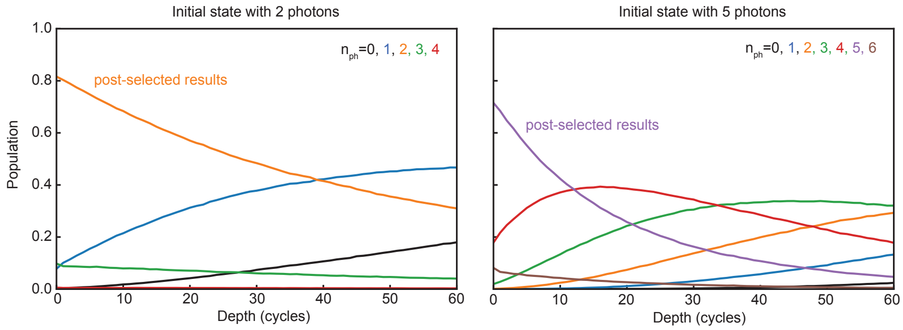

FIG. S6. **Post-selection for Figure 2:** In the trajectory experiments shown in the main text (Figures 2 and 4), we post select the outcome bitstrings that preserve the number of photons. This post-selection is justified by the fact that the fSim gate is an excitation preserving gate for any angle. The observed decay is due to the T_1 decay of the qubits during the circuit. The measurement becomes more susceptible to T_1 errors as the size of the bound state increases, thus increasing the necessary number of repetitions to construct the statistics.

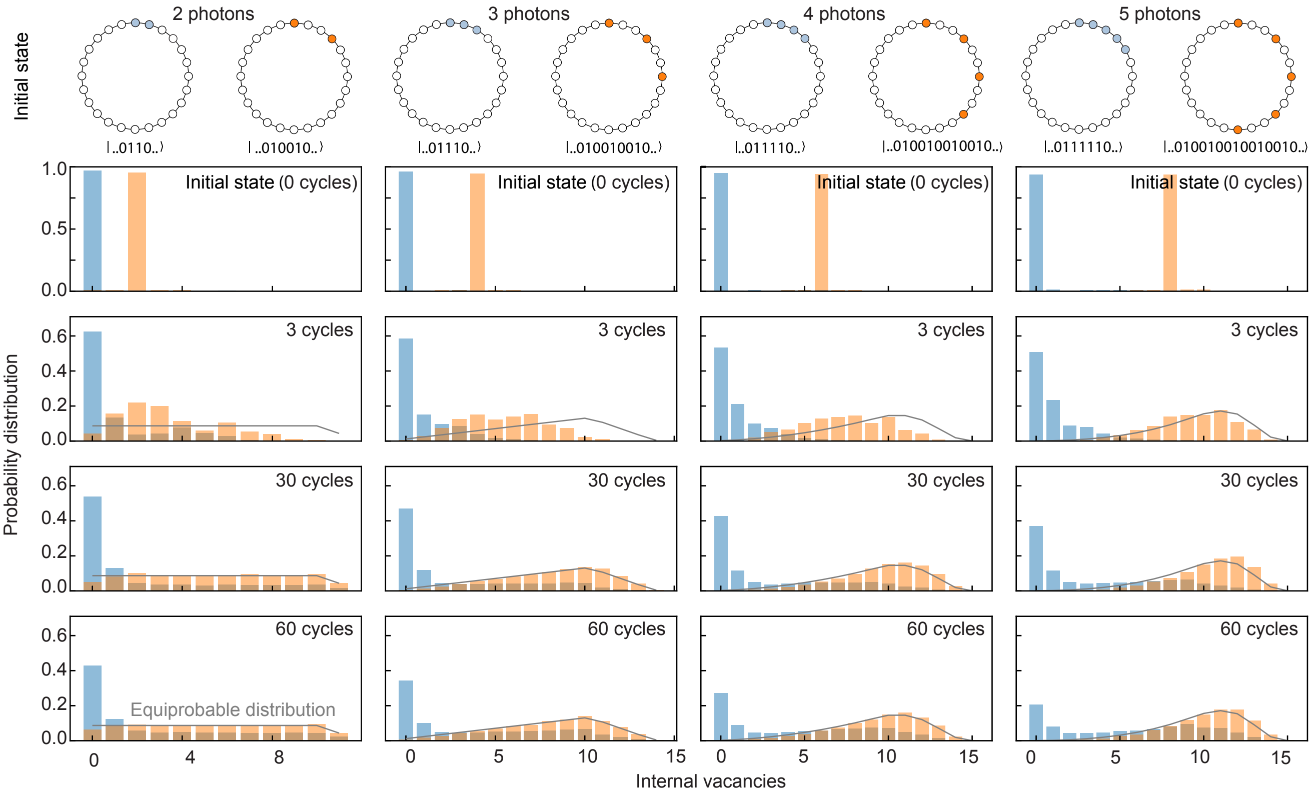

FIG. S7. **Trajectory histogram:** Complementary data for Figure 2 in the main text with angles $\theta = \pi/6$ and $\phi = 2\pi/3$. For each number of photons, we initialize the system with adjacent excitations (blue) or with excitations separated by a few vacancies (orange). In the case where the excitations are initially adjacent, we find that the distribution stays concentrated in the part of Hilbert space with few vacancies. When the initial state contains vacancies, on the other hand, the evolution tends toward exploring the entire Hilbert space and approaches the equiprobable distribution represented by a dashed line.

B. Spectroscopy

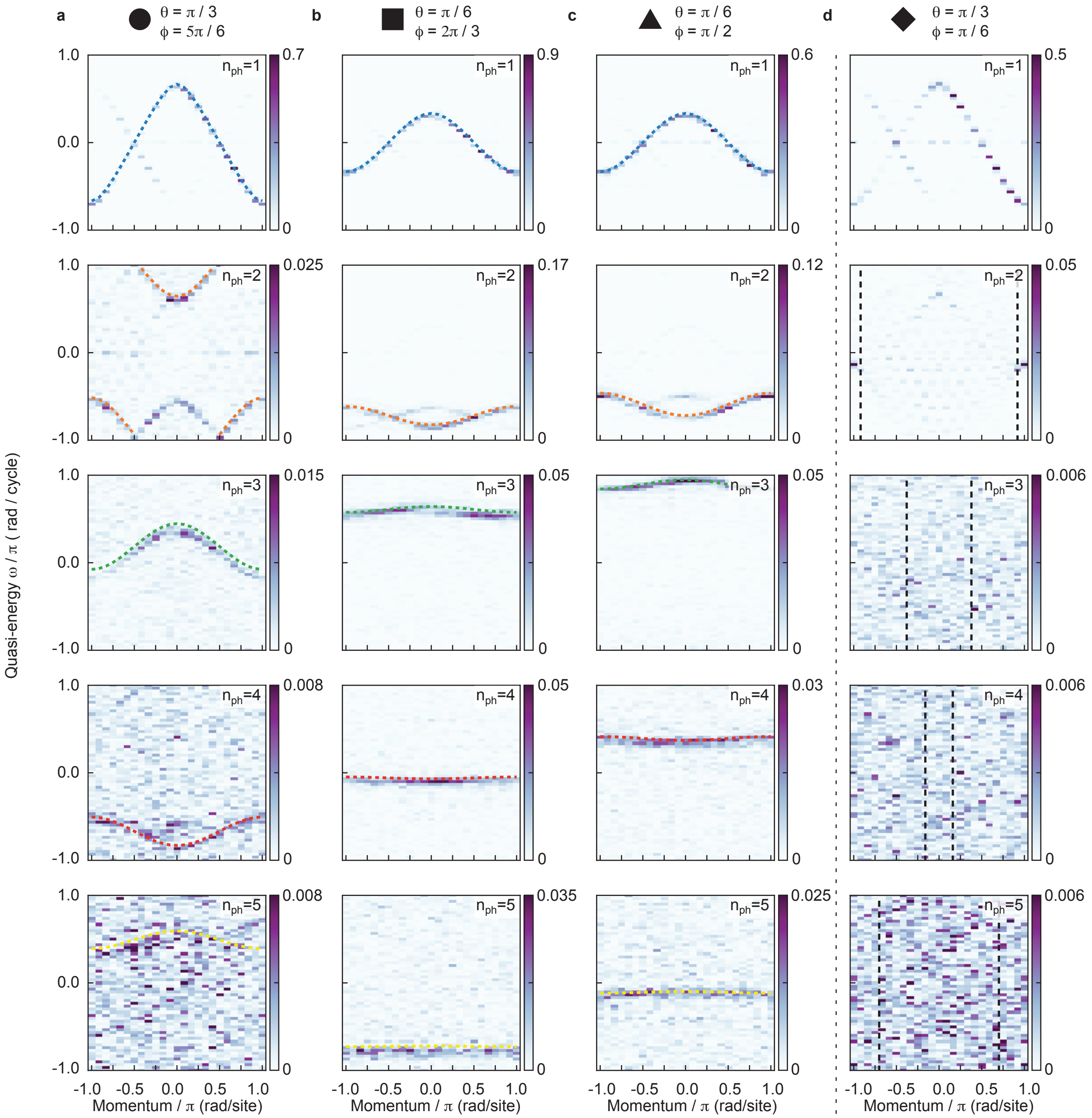

FIG. S8. **Many-body spectroscopy for various fSim angles. a**, Band structures analogous to those shown in Fig. 3c in the main text for $n_{\mathrm{ph}} = 1-5$ (top to bottom) and fSim angles $\theta = \pi/3$, $\phi = 5\pi/6$ (**a**), $\theta = \pi/6$, $\phi = 2\pi/3$ (**b**), $\theta = \pi/6$, $\phi = \pi/2$ (**c**), $\theta = \pi/3$, $\phi = \pi/6$ (**d**). Dashed lines: theoretical prediction. We observe very good agreement with the theoretically predicted band structure across all the three angle pairs that satisfy $\phi > 2\theta$, required to have bound states at all momenta. As predicted by theory, the bands are found to shift with increasing interaction strength (ϕ), and the width of the dispersion increases with increasing θ. In the regime where $\phi < 2\theta$, we observe a two-photon bound state for momenta near $k = \pm\pi$, while no bound states are observed for higher n_{ph} in this regime. The latter is likely due to the fact that the overlap between the initial product state and the n_{ph}-bound state scales as $2^{-n_{\mathrm{ph}}}$, thus causing a reduction in the signal-to-noise ratio at high n_{ph}. Dashed black vertical lines: theoretically predicted momentum threshold for the existence of bound states.

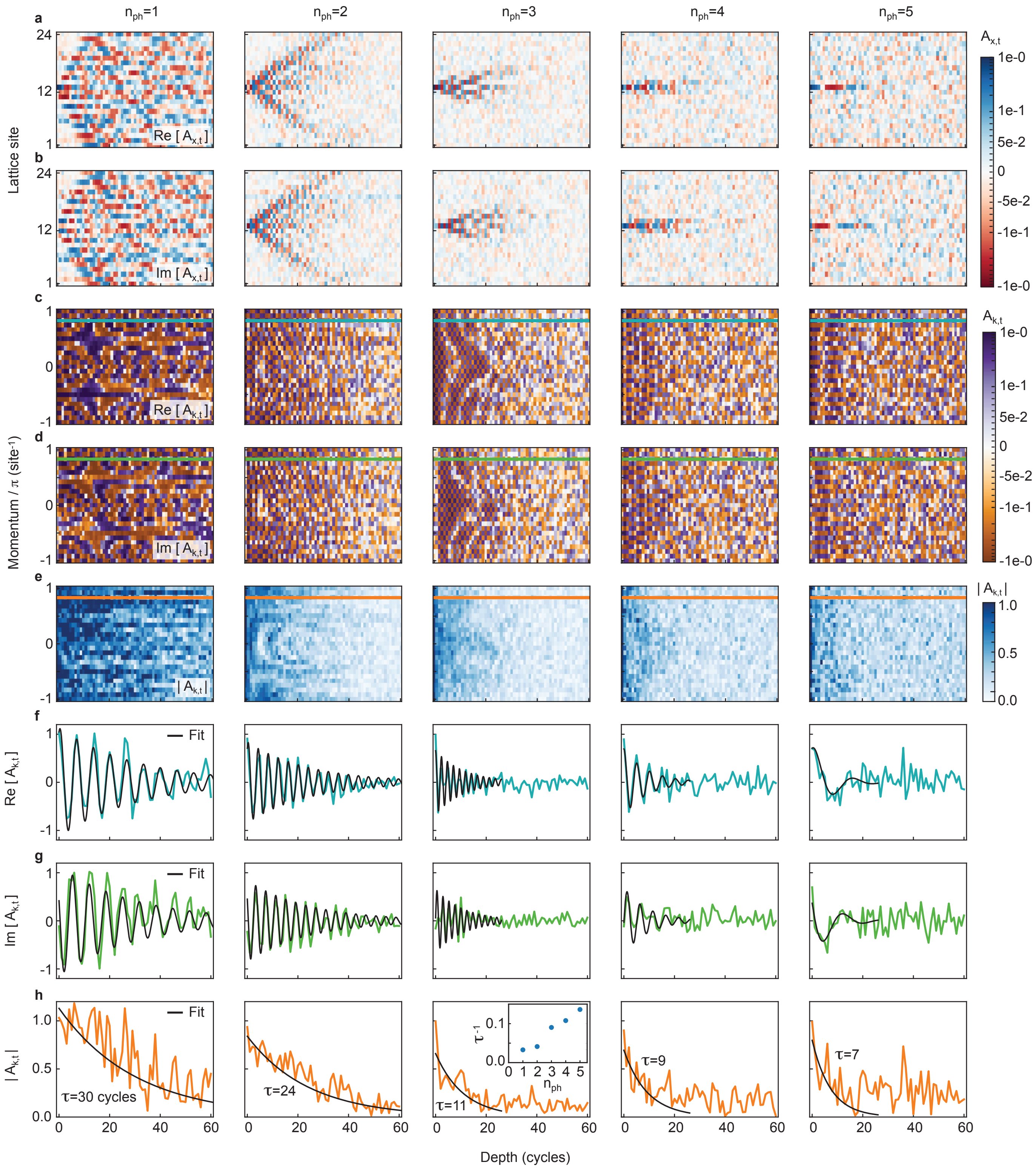

FIG. S9. **Decoherence of multi-particle correlator.** **a,b**, Real (**a**) and imaginary (**b**) parts of the correlator $\langle C_{j,n_{\mathrm{ph}}}\rangle = \langle \Pi_{i=j}^{j+n_{\mathrm{ph}}-1}\sigma_i^+\rangle$ for $n_{\mathrm{ph}} = 1-5$. **c,d**, Same as **a,b**, but Fourier transformed to momentum space. **e**, Absolute value of the multi-particle correlator Fourier transformed to momentum space. **f-h**, Linecuts at $k = 5\pi/6$ for **c**, **d**, and **e**, respectively. Black curves show fits on the form $A_{k=5\pi/6,t} = \alpha e^{(i\omega-1/\tau)t}$ (single fit for all three plots at each photon number). Inset in center panel: extracted decay rate τ^{-1} (in units of cycles^{-1}) as a function of photon number.

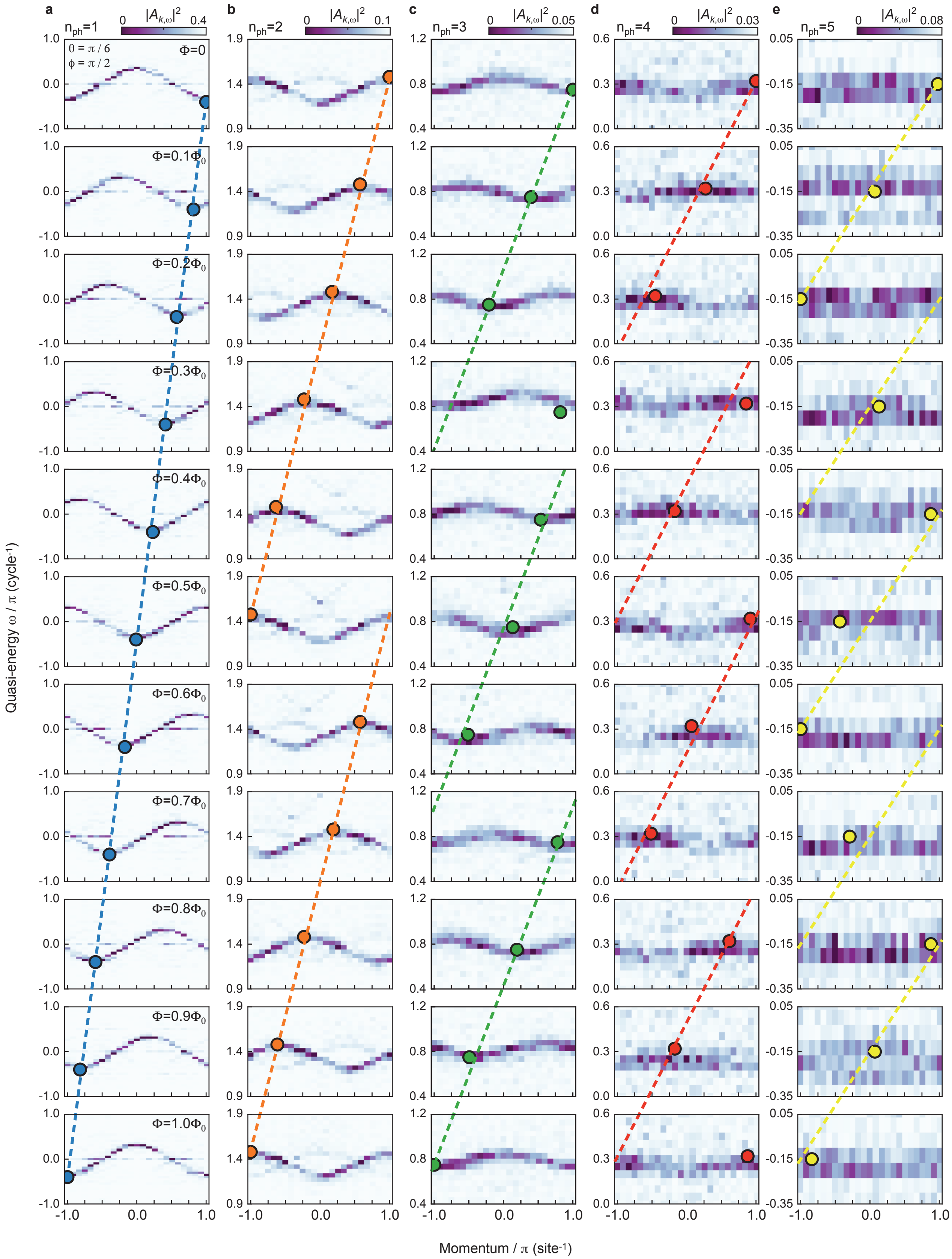

FIG. S10. **Flux dependence of band structures** for $n_{\rm ph} = 1 - 5$ (**a-e**, respectively). Momentum shifts were extracted by convolving the band structures with that at $\Phi = 0$ ($\Phi = 0.2\Phi_0$ for $n_{\rm ph} = 5$ due to more clear structure), summing over the energy axis, and finding the maximum. Colored dots indicate the corresponding extracted peak positions of the bands.

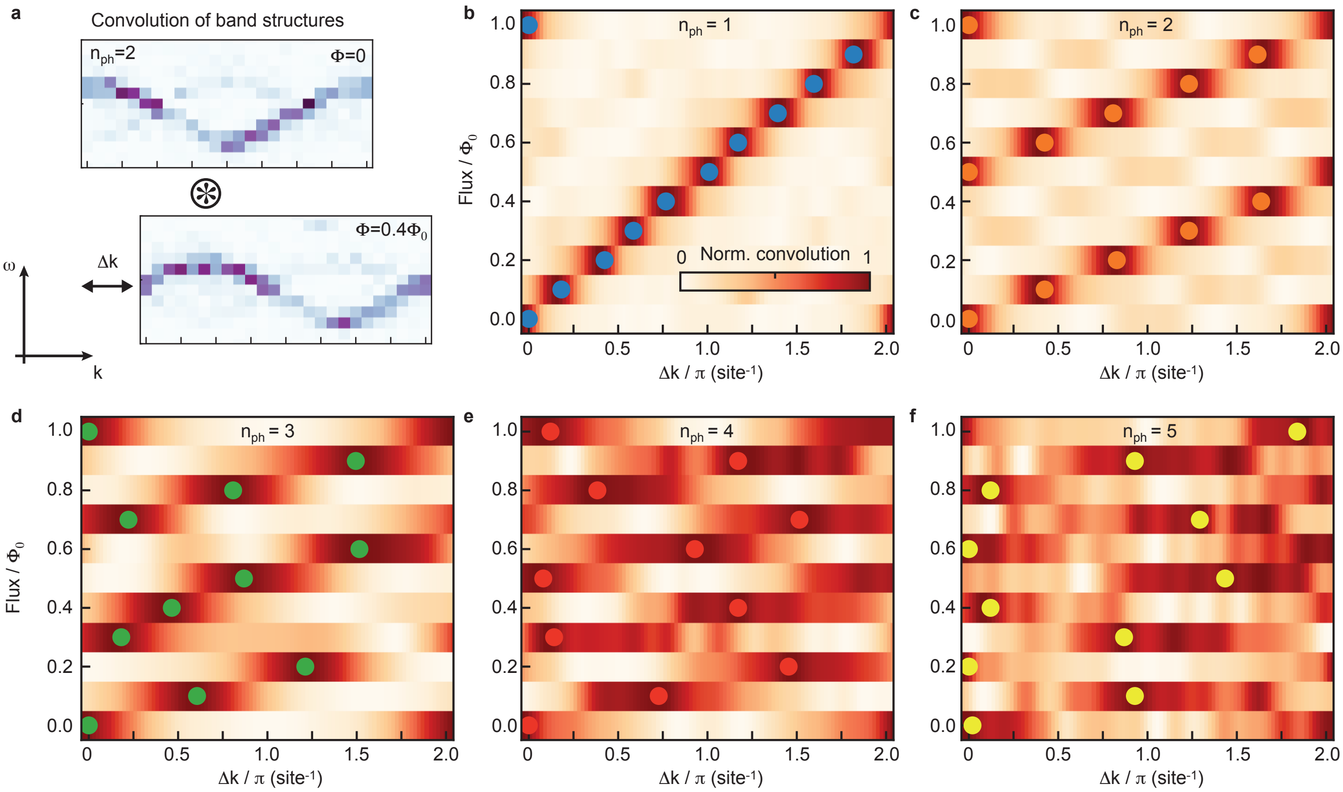

FIG. S11. **Convolution technique for extracting momentum shifts.** **a**, The flux-induced momentum shifts shown in Fig. 3e,f in the main text are determined through cyclic convolution of the band structures ($|A_{k,\omega}|^2$) with that at $\Phi = 0$ ($\Phi = 0.2\Phi_0$ for $n_{\text{ph}} = 5$ due to more clear structure), followed by summation over the energy axis. The shift is then found from the maximum of the convolution. The signal-to-noise ratio is maximized by only including energies in the vicinity of the band. Moreover, in order to extract momentum shifts with a finer resolution than $2\pi/24$ site^{-1}, the band structures are interpolated along the momentum direction before convolution, increasing the number of points from 24 to 100. **b-f**, Normalized convolutions for $n_{\text{ph}} = 1 - 5$ for the full flux range ($\Phi = 0 - \Phi_0$). Each row is normalized relative to its respective extrema, such that their ranges span from 0 to 1. Colored dots indicate the maxima at each flux value.

VI. Integrability of the Floquet XXZ model

The Floquet XXZ model was solved in [6] using the coordinate Bethe ansatz. The possibility of such a solution follows from the results of [7] where the relation of the unitary matrix of the Floquet evolution U_F to the family of transfer matrices $\hat{T}(\lambda) : [\hat{T}(\lambda_1), \hat{T}(\lambda_2)] = 0$ was established. The purpose of this section is to re-derive integrability in a somewhat different fashion to make a connection with the explicit form of the fSim gates. Let us recall the starting point of the derivation (Eq (12) of Ref. [6]):

$$\hat{\mathcal{U}} = \hat{t}^2[\hat{\mathcal{V}}]^2, \tag{S30}$$

where $\hat{t}^2$ is the translation operator commuting with all the other operators, and the relation between $\hat{\mathcal{U}}, \hat{\mathcal{V}}$ are shown in Fig. S12a and b respectively. The single node is the fSim gate given with the notation of [6] as:

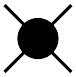

$$= \text{fSim}\,(\theta, \phi) = \begin{pmatrix} 1 & 0 & 0 & 0 \\ 0 & \cos\theta & i\sin\theta & 0 \\ 0 & i\sin\theta & \cos\theta & 0 \\ 0 & 0 & 0 & e^{2i\phi} \end{pmatrix} \tag{S31}$$

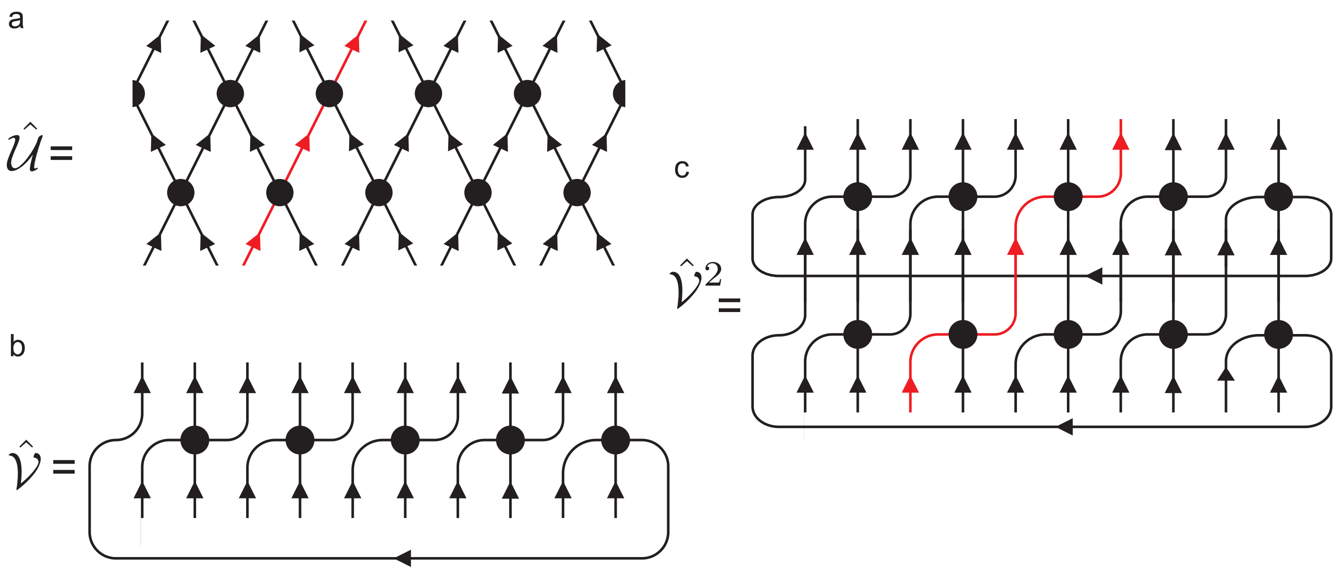

FIG. S12. **Digramatic representation.** **a**, Graphic representation of the original model. Each node represents an fSim gate. The half dot on the edge of the figure represents the periodicity of the system. **b**, Graphic representation of a simpler model. **c**, $\hat{\mathcal{V}}^2$ is similar to the original model up to a translation.

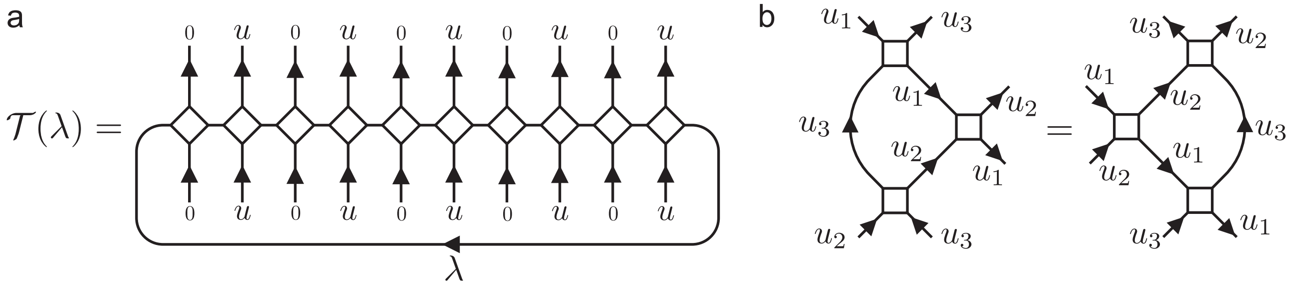

FIG. S13. **Digramatic representation.** **a**:general transfer matrix $\hat{T}(\lambda)$. **b**: representation of the Yang-Baxter relation.

The integrability of this model is not easy to see in the original matrix $\hat{\mathcal{U}}$. In constrast, one can notice that elementary steps in the matrix $\hat{\mathcal{V}}$ involves jumps only by one qubit that makes its diagonalization much easier. The

explicit integration of the matrix $\hat{\mathcal{V}}$ is done in [6] by coordinate Bethe ansatz. In what follows, we illustrate how the notion of integrability appears in terms of the Algebraic Bethe Ansatz (see e.g. ch. 2 of [8]). To this end, let us introduce the more general transfer matrix $\hat{T}(\lambda)$ defined in Fig. S13a.

$[\hat{T}(\lambda_1), \hat{T}(\lambda_2)] = 0$ for any λ_1, λ_2 if the Yang-Baxter relation is satisfied (Fig S13b). We then notice that:

$$\hat{\mathcal{V}} = \exp\Big(i\phi\hat{\mathcal{N}_z}\Big)\hat{T}(0), \tag{S32}$$

where $\hat{\mathcal{N}_z}$ is the conserved number of excitations in the system, $[\hat{\mathcal{N}_z}, \hat{T}(\lambda)] = 0$, that does not affect any commutation relation. The previous equation is valid provided that we have the two following equalities:

0
0
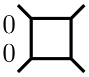
$=$
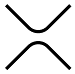

$$= \begin{pmatrix} 1 & 0 & 0 & 0 \\ 0 & 0 & 1 & 0 \\ 0 & 1 & 0 & 0 \\ 0 & 0 & 0 & 1 \end{pmatrix}; \tag{S33}$$

and

u
0
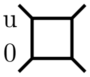

$$= \begin{pmatrix} 1 & 0 & 0 & 0 \\ 0 & e^{-i\phi}\cos\theta & ie^{-i\phi}\sin\theta & 0 \\ 0 & ie^{-i\phi}\sin\theta & e^{-i\phi}\cos\theta & 0 \\ 0 & 0 & 0 & 1 \end{pmatrix} \tag{S34}$$

The Baxter-Yang equation and the previous equation were resolved in [7] with:

u_1
u_2
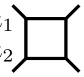

$$= \begin{pmatrix} 1 & 0 & 0 & 0 \\ 0 & \frac{\sin u_{12}}{sin(u_{12}+i\eta)} & \frac{\sin(i\eta)}{sin(u_{12}+i\eta)} & 0 \\ 0 & \frac{\sin(i\eta)}{sin(u_{12}+i\eta)} & \frac{\sin u_{12}}{sin(u_{12}+i\eta)} & 0 \\ 0 & 0 & 0 & 1 \end{pmatrix} \tag{S35}$$

with $u_{12} = u_2 - u_1$. The matrix satisfies the equation of Fig. S13b as being the canonical form of the six vertex model intertwiner, whereas Eq. S34 fixes the undetermined and so far independent parameters u, η. (They are nothing but that given in Eq. (63c) of Ref. [6] with $u = \alpha$).

To summarize, the equations given in this section guarantee the integrability of the problem. Vice versa, the fact that the explicit Bethe Ansatz of [6] diagonalizes the Floquet evolution implies the existence of an infinite number of commuting operators of $\Big[\hat{T}(\lambda_1), \hat{T}(\lambda_2)\Big] = 0$ [8].

VII. Numerical analysis of the breaking of integrability

A. Level statistics

In this section we numerically verify the chaotic nature of the perturbed model by analysing the level statistics of the Floquet unitary for the system parameters used in experiment. To perform the analysis, we first extract the eigenvalues of the Floquet unitary $\hat{U}_F$ defined in Figure 4b of the main text, $\mathcal{E}(\hat{U}_F) = \{e^{i\chi_i}\}, \quad \chi_i \in [-\pi, \pi), \quad \chi_{i+1} \geq \chi_i$. We consider states with 3 and 4 photons. Since the system is invariant under translations by two sites, we restrict our attention to the zero momentum sector. To characterize the level statistics we calculate the ratios of consecutive phase gaps,

$$r_i = \frac{\min(\delta_i, \delta_{i+1})}{\max(\delta_i, \delta_{i+1})}, \tag{S36}$$

where $\delta_i = \chi_i - \chi_{i-1} (\mathrm{mod}\ 2\pi)$. The relevant random matrix distribution is the circular orthogonal ensemble (COE), because even though $\hat{U}_F \neq \hat{U}_F^T$, $\mathcal{E}(\hat{U}_F) = \mathcal{E}(\hat{U}_F^T)$, since the two unitaries are related by a canonical transformation. The resulting distribution of the parameter r is illustrated in Fig. S14. We observe that for a moderate value of the integrability breaking parameter, the distribution is approximately consistent with the COE statistics, indicating that the system's integrability is indeed broken.

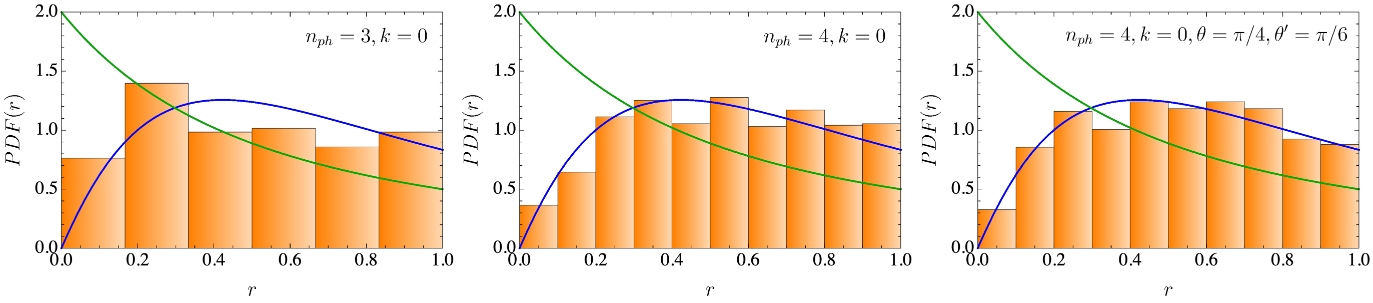

FIG. S14. Probability distribution of the spectral parameter r (as defined in Eq. S36) for the Floquet operator $\hat{U}_F$. Left and central panels are for the experimental system parameters $\phi = \phi' = 2\pi/3, \theta = \pi/6$, and for $\theta' = \pi/8$. The green line displays the Poisson distribution of energy levels, $P_P = 2/(1+r)^2$, and the blue line shows the COE distribution [9]. The Hilbert space dimensions are $\mathcal{D}_{n_{ph}=4,k=0} = 855$ and $\mathcal{D}_{n_{ph}=3,k=0} = 190$. Despite the small Hilbert space sizes, we observe that the integrability-breaking perturbation induces a strong level repulsion. The distribution averages for the experimental parameters are $\langle r \rangle_4 \sim 0.544, \langle r \rangle_3 \sim 0.5$, thus illustrating the proximity to COE statistics, which is characterized by $\langle r \rangle_{\mathrm{COE}} \sim 0.527$. A Poisson distribution, on the other hand, corresponds to $\langle r \rangle_P \sim 0.386$. Right panel: distribution of the r-parameter for a larger swap angle, $\theta = \pi/4$. Similarly, a moderate integrability breaking parameter $\theta' = \pi/6$ leads to level repulsion and level statistics consistent with COE.

B. Stability of the $n_{\mathrm{ph}} = 3$ domain wall

In this section we numerically explore the stability of the bound state of $n_{\mathrm{ph}} = 3$ photons (which we here also refer to as the domain wall state). We present simulations both for the experimental system parameters, where we employ exact diagonalization, and for a much larger systems using matrix-product states methods. In Figure S15a we show the stability of the bound states for the parameters identical to those in the experimental setup (coherent system of 24 qubits) at longer times and different values of the perturbation angle θ'. In the simulation we place three excitations at sites Q_1, Q_2, Q_3 (see Figure 4b of main text), and the system is evolved using the same unitary $\hat{U}_F$. The domain wall decay is probed by measuring the probability that the system remains in the subspace with three adjacent excitations. Note that this subspace includes configurations with three adjacent excitations in the original chain, as well as configurations where one excitation is located in one of the additional qubits coupled to the ring. The resulting behavior indicates that the survival probability remains finite, and therefore the bound states remain stable up to extremely long times for a broad range of θ' values. Thus, the observed robustness of the bound states is not limited to the shorter time scales that are accessible experimentally.

Further, in Figure S16 we employ the matrix-product state (MPS) ansatz and time-evolving-block-decimation algorithm (TEBD) [10] to explore the decay of the domain wall states in the thermodynamic limit $L \to \infty$. In practice we use an open finite system of length $L = 800$ and simulate the quench up to time $T = 400$. We initialize the three

excitations at the centre of the chain $(L/2-1, L/2, L/2+1)$, such that the perturbation is coupled to the sites at positions $L/2 \pm 1$. For times $t \leq T$ the Lieb-Robinson bound [11] guarantees that the information propagation from the centre of the chain will not have reached the boundaries, and therefore, the bound state propagates without any boundary effects. The domain wall state survival is again probed by measuring the probability of the system to remain in the subspace with three adjacent excitations.

For the experimental parameters $\theta = \pi/6, \phi = 2\pi/3$ (right panel in Fig. S16), we observe that up to sizeable values of the integrability-breaking perturbation $\theta' \lesssim \pi/4$, the survival probability appears to saturate at a finite value at times $t = 400$, indicating the robustness of the bound states in large systems. Only at large values of the perturbation θ', the survival probability exhibits a slow decay at large times $150 \lesssim t \lesssim 400$ that appears to be consistent with an exponential fit. Further work is needed to understand the physical mechanism of the very slow decay of the bound states. Unexpectedly, in contrast to the case $\theta = \pi/6$, at larger value of $\theta = \pi/4$ and $\phi = 2\pi/3$ (left panel of Fig. S16), the survival probability decays at any value of θ', indicating the diminished robustness of the bound states. For these values of θ and ϕ the observed behavior is consistent with an exponential decay at $t \gtrsim 150$. We have also studied numerically the effect of other perturbations of the integrable dynamics: we added different Z rotations on even and odd sites, allowed for different rotation angles $\theta_{i,i+1}$ or $\phi_{i,i+1}$ for even and odd i. We observed that all these perturbations have remarkably little effect on the stability of the bound states. The only exception is the addition of X rotation that violates the particle conservation. This perturbation has indeed large effect on the bound states.

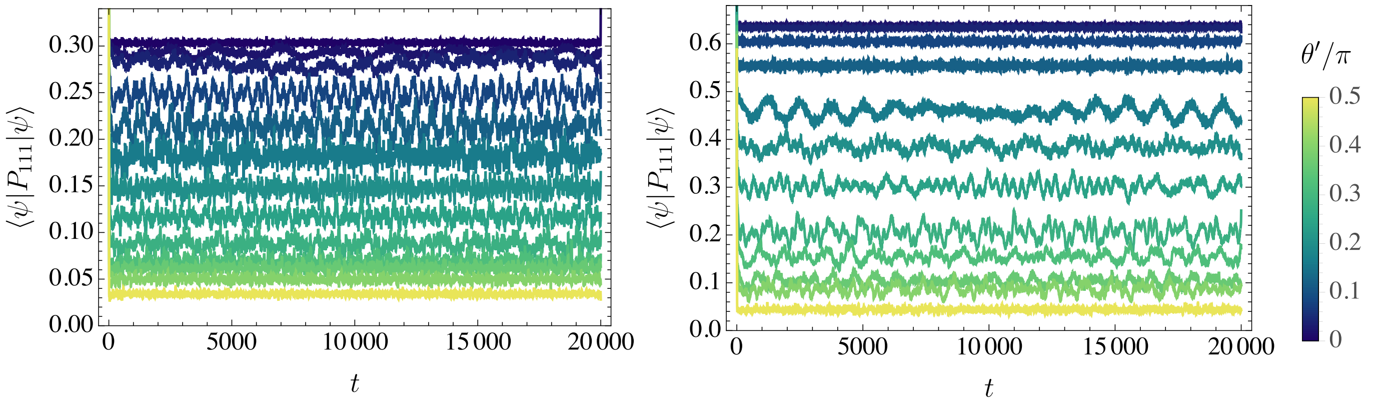

FIG. S15. Decay of the $n_{ph} = 3$ domain-wall state for the experimental setup for $\phi = \phi' = 2\pi/3$ and $\theta = \pi/4$ (left), $\theta = \pi/6$ (right). In both cases we observe that the probability of finding three adjacent excitations saturates to a finite value at long times for a broad regime of integrability-breaking perturbations θ', indicating robustness of the bound states. Values of the integrability-breaking perturbation are $\theta'/\pi = 0, 0.02, 0.04, 0.08, 0.12, 0.16, ...0.36, 0.4, 0.5$ (top to bottom curves).

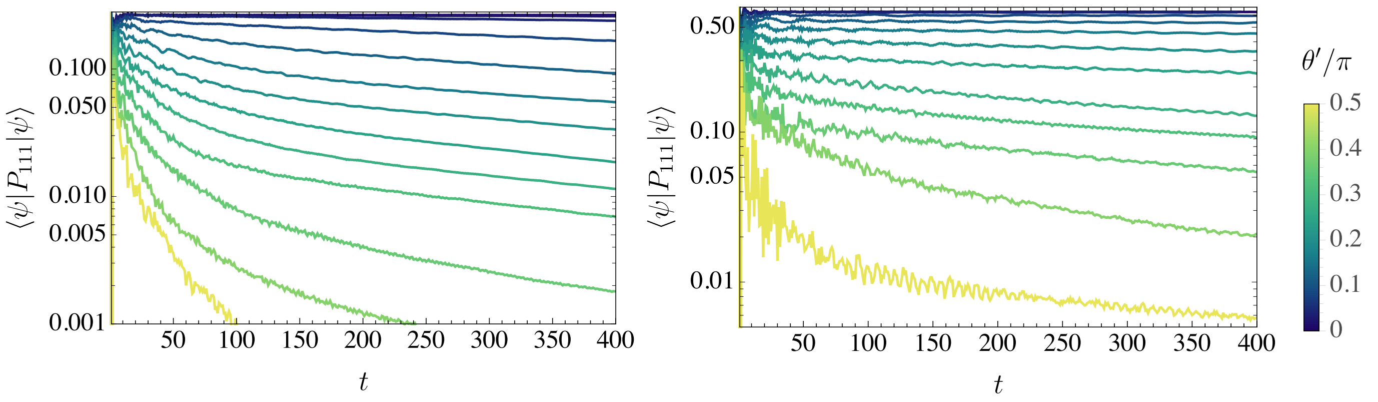

FIG. S16. Decay of the $n_{ph} = 3$ domain-wall state for a system of length $L = 800$ and parameters $\phi = \phi' = 2\pi/3$ and $\theta = \pi/4$ (left), $\theta = \pi/6$ (right), plotted in linear-logarithmic scale. Values of the integrability-breaking perturbation are $\theta'/\pi = 0, 0.02, 0.04, 0.08, 0.12, 0.16, ...0.36, 0.4, 0.5$ (top to bottom curves).

† Google Quantum AI and Collaborators

A. Morvan[1,‡], T. I. Andersen[1,‡], X. Mi[1,‡], C. Neill[1,‡], A. Petukhov[1], K. Kechedzhi[1], D. A. Abanin[1, 2], A. Michailidis[2] R. Acharya[1], F. Arute[1], K. Arya[1], A. Asfaw[1], J. Atalaya[1], R. Babbush[1], D. Bacon[1], J. C. Bardin[1, 3], J. Basso[1], A. Bengtsson[1], G. Bortoli[1], A. Bourassa[1], J. Bovaird[1], L. Brill[1], M. Broughton[1], B. B. Buckley[1], D. A. Buell[1], T. Burger[1], B. Burkett[1], N. Bushnell[1], Z. Chen[1], B. Chiaro[1], R. Collins[1], P. Conner[1], W. Courtney[1], A. L. Crook[1], B. Curtin[1], D. M. Debroy[1], A. Del Toro Barba[1], S. Demura[1], A. Dunsworth[1], D. Eppens[1], C. Erickson[1], L. Faoro[1], E. Farhi[1], R. Fatemi[1], L. Flores Burgos[1] E. Forati[1], A. G. Fowler[1], B. Foxen[1], W. Giang[1], C. Gidney[1], D. Gilboa[1], M. Giustina[1], A. Grajales Dau[1], J. A. Gross[1], S. Habegger[1], M. C. Hamilton[1], M. P. Harrigan[1], S. D. Harrington[1], J. Hilton[1], M. Hoffmann[1], S. Hong[1], T. Huang[1], A. Huff[1], W. J. Huggins[1], S. V. Isakov[1], J. Iveland[1], E. Jeffrey[1], Z. Jiang[1], C. Jones[1], P. Juhas[1], D. Kafri[1], T. Khattar[1], M. Khezri[1], M. Kieferová[1, 8], S. Kim[1], A. Y. Kitaev[1, 5], P. V. Klimov[1], A. R. Klots[1], A. N. Korotkov[1, 6], F. Kostritsa[1], J. M. Kreikebaum[1], D. Landhuis[1], P. Laptev[1], K.-M. Lau[1], L. Laws[1], J. Lee[1], K. W. Lee[1], B. J. Lester[1], A. T. Lill[1], W. Liu[1], A. Locharla[1], E. Lucero[1], F. Malone[1], O. Martin[1], J. R. McClean[1], M. McEwen[1, 7], B. Meurer Costa[1], K. C. Miao[1], M. Mohseni[1], S. Montazeri[1], E. Mount[1], W. Mruczkiewicz[1], O. Naaman[1], M. Neeley[1], A. Nersisyan[1], M. Newman[1], A. Nguyen[1], M. Nguyen[1], M. Y. Niu[1], T. E. O'Brien[1], R. Olenewa[1], A. Opremcak[1], R. Potter[1], C. Quintana[1], N. C. Rubin[1], N. Saei[1], D. Sank[1], K. Sankaragomathi[1], K. J. Satzinger[1], H. F. Schurkus[1], C. Schuster[1], M. J. Shearn[1], A. Shorter[1], V. Shvarts[1], J. Skruzny[1], W. C. Smith[1], D. Strain[1], G. Sterling[1], Y. Su[1], M. Szalay[1], A. Torres[1], G. Vidal[1], B. Villalonga[1], C. Vollgraff-Heidweiller[1], T. White[1], C. Xing[1], Z. Yao[1], P. Yeh[1], J. Yoo[1], A. Zalcman[1], Y. Zhang[1], N. Zhu[1], H. Neven[1], S. Boixo[1], A. Megrant[1], J. Kelly[1], Y. Chen[1], V. Smelyanskiy[1], I. Aleiner[1,§], L. B. Ioffe[1,§], P. Roushan[1,§]

1 Google Research, Mountain View, CA, USA
2 Department of Theoretical Physics, University of Geneva, Quai Ernest-Ansermet 30, 1205 Geneva, Switzerland
3 Department of Electrical and Computer Engineering, University of Massachusetts, Amherst, MA, USA
4 Department of Applied Physics, Yale University, New Haven, CT 06520, USA
5 Institute for Quantum Information and Matter, California Institute of Technology, Pasadena, CA, USA
6 Department of Electrical and Computer Engineering, University of California, Riverside, CA, USA
7 Department of Physics, University of California, Santa Barbara, CA, USA
8 Centre for Quantum Computation and Communication Technology, Centre for Quantum Software and Information, Faculty of Engineering and Information Technology, Univ

‡ These authors contributed equally to this work.
§ Corresponding author: igoraleiner@google.com
§ Corresponding author: ioffel@google.com
§ Corresponding author: pedramr@google.com

---

[1] F. Arute, K. Arya, R. Babbush, D. Bacon, J. C. Bardin, *et al.*, Quantum supremacy using a programmable superconducting processor, Nature **574**, 505 (2019).
[2] C. Neill, T. McCourt, X. Mi, Z. Jiang, M. Y. Niu, *et al.*, Accurately computing the electronic properties of a quantum ring, Nature **594**, 508 (2021).
[3] F. Arute, K. Arya, R. Babbush, D. Bacon, J. C. Bardin, *et al.*, Observation of separated dynamics of charge and spin in the fermi-hubbard model (2020).
[4] X. Mi, M. Ippoliti, C. Quintana, A. Greene, Z. Chen, *et al.*, Time-crystalline eigenstate order on a quantum processor, Nature **601**, 531 (2022).
[5] B. Foxen, C. Neill, A. Dunsworth, P. Roushan, B. Chiaro, *et al.* (Google AI Quantum), Demonstrating a continuous set of two-qubit gates for near-term quantum algorithms, Phys. Rev. Lett. **125**, 120504 (2020).
[6] I. L. Aleiner, Bethe ansatz solutions for certain periodic quantum circuits, Annals of Physics **433**, 168593 (2021).
[7] M. Ljubotina, L. Zadnik, and T. Prosen, Ballistic spin transport in a periodically driven integrable quantum system, Phys. Rev. Lett. **122**, 150605 (2019).
[8] C. Gómez, M. Ruiz-Altaba, and G. Sierra, *Quantum Groups in Two-Dimensional Physics* (1996).
[9] L. D'Alessio and M. Rigol, Long-time behavior of isolated periodically driven interacting lattice systems, Phys. Rev. X **4**, 041048 (2014).
[10] G. Vidal, Efficient classical simulation of slightly entangled quantum computations, Phys. Rev. Lett. **91**, 147902 (2003).
[11] E. Lieb and D. Robinson, The finite group velocity of quantum spin systems, Communications in Mathematical Physics **28**, 251 (1972).
